# Supplementary material for: Diagnostic Algorithm for Surgical Management of Limbal Stem Cell Deficiency
Source: Diagnostics (Basel). 2023 Jan 5;13(2):199. doi: 10.3390/diagnostics13020199 (PMC9858342; doi:10.3390/diagnostics13020199)
Supplement: Supplementary file 1 [file diagnostics-13-00199-s001.zip › diagnostics-2062204-supplementary.pdf]

Supplementary Table S1. Various cytokine levels in the tear samples pre- 3, 6 and 12 months postoperatively (after GSLET) in patients with partial and total LSCD (pg/mL).

Cytokine	Partial LSCD (n=5)				Total LSCD (n=6)			
	**before**	**3m**	**6m**	**12m**	**before**	**3m**	**6m**	**12m**
IL-8	162.32±79.90 (p=0.9998)	124.00±29.50 (p=0.9829)	47.65±13.73 (p=0.0011)	30.80±10.03 (p=0.041)	158.80±24 (p=0.9998)	115.22±25.25 (p=0.9829)	104.93±2.60 (p=0.0011)	70.93±15.40 (p=0.041)
IL-4	78.17±20.95 <0.0001	51.49±19.05 (p<0.0001)	19.82±3.57 (p<0.0001)	17.37±5.91 (p=0.9536)	34.58±6.07 (p<0.0001)	80.01±8.72 (p<0.0001)	69.26±2.33 (p<0.0001)	21.46±3.64 (p=0.9536)
IL-1β	93.20±14.52 (p=0.1035)	57.27±50.47 (p<0.0001)	10.11±3.59 (p=0.981)	6.00±1.76 (p=0.9462)	128.00±57.88 (p=0.1035)	173.51±27.71 (p<0.0001)	19.10±2.88 (p=0.981)	17.48±5.37 (p=0.9462)
IL-10	27.16±5.09 (p=0.007)	39.65±17.51 (p<0.0001)	13.70±2.98 (p=0.2008)	3.28±1.26 (p=0.991)	41.96±11.99 (p=0.007)	84.85±21.74 (p<0.0001)	22.88±5.43 (p=0.2008)	5.59±2.27 (p=0.991)
TGF-β	1642.24±976.47 (p<0.0001)	619.05±303.36 (p=0.9942)	92.19±36.28 (p=0.0108)	64.87±39.64 (p=0.9997)	465.89±106.56 (p<0.0001)	524.63±88.78 (p=0.9942)	733.65±72.80 (p=0.0108)	116.28±50.13 (p=0.9997)
TNF-α	136.80±22.22 (p<0.0001)	381.11±96.85 (p=0.0031)	106.48±37.42 (p<0.0001)	79.61±31.11 (p=0.2481)	411.16±102.31 (p<0.0001)	501.60±52.43 (p=0.0031)	479.70±68.71 (p<0.0001)	145.60±49.28 (p=0.2481)

Abbreviations: LSCD – limbal stem cell deficiency, IL – interleukin, TGF – transforming growth factor, TNF – tumor necrosis factor, pg/mL – picograms per milliliter, p - mean square deviation. Data presented as mean±standard deviation.

Supplementary Table S2. Correlation analysis of cytokines in the preoperative period in patients with partial and total LSCD (pg/mL).

Cytokine	Partial LSCD (n=5)						Cytokine	Total LSCD (n=6)					
	IL-8	IL-4	IL-1β	IL-10	TGF-β	TNF-α		IL-8	IL-4	IL-1β	IL-10	TGF-β	TNF-α
IL-8		0.74 (p=0.014)	-0.25 (p=0.489)	0.04 (p=0.918)	0.45 (p=0.191)	0.32 (p=0.363)	IL-8		1.00 (p=0.000)	0.35 (p=0,326)	-0.98 (p=0.000)	-0.04 (p=0.910)	-0.23 (p=0.523)

IL-4	0.74 (p=0.014)		0.08 (p=0.833)	0.15 (p=0.683)	0.68 (p=0.032)	0.02 (p=0.962)	IL-4	1.00 (p=0.000)		0.35 (p=0,316)	-0.99 (p=0.000)	-0.03 (p=0.927)	-0.21 (p=0.561)
IL-1β	-0.25 (p=0.489)	0.08 (p=0.833)		-0.07 (p=0.846)	-0.43 (p=0.213)	0.11 (p=0.630)	IL-1β	0.35 (p=0.326)	0.35 (p=0.316)		-0.37 (p=0.297)	0.57 (p=0.086)	-0.23 (p=0.523)
IL-10	0.04 (p=0.918)	0.15 (p=0.683)	-0.07 (p=0.846)		0.18 (p=0.610)	0.11 (p=0.761)	IL-10	-0.98 (p=0.000)	-0.99 (p=0.000)	-0.37 (p=0.297)		0.01 (p=0.978)	0.14 (p=0.690)
TGF-β	0.45 (p=0.191)	0.68 (p=0.032)	-0.43 (p=0.213)	0.18 (p=0.610)		-0.10 (p=0.782)	TGF-β	-0.04 (p=0.910)	-0.03 (p=0.927)	0.57 (p=0.086)	0.01 (p=0.978)		-0.15 (p=0.683)
TNF-α	0.32 (p=0.363)	0.02 (p=0.962)	0.17 (p=0,630)	0.11 (p=0.761)	-0.10 (p=0.782)		TNF-α	-0.23 (p=0.523)	-0.21 (p=0,561)	-0.23 (p=0.523)	0.14 (p=690)	-0.15 (p=0.683)	

Supplementary Table S3. Correlation analysis of cytokines 3 months postoperatively (after GSLET) in patients with partial and total LSCD (pg/mL).

**Cytokine**	**Partial LSCD (n=5)**						**Cytokine**	**Total LSCD (n=6)**					
	IL-8	IL-4	IL-1β	IL-10	TGF-β	TNF-α		IL-8	IL-4	IL-1β	IL-10	TGF-β	TNF-α
IL-8		0.99 (p=0.000)	0.92 (p=0.000)	0.35 (p=0.324)	0.98 (p=0.000)	0.74 (p=0.015)	IL-8		-0.30 (p=0.395)	0.07 (p=0.838)	-0.27 (p=0.455)	0.25 (p=0.493)	-0.28 (p=0.440)
IL-4	0.99 (p=0.000)		0.90 (p=0.000)	0.31 (p=0.386)	0.99 (p=0.000)	0.68 (p=0.030)	IL-4	-0.30 (p=0.395)		0.00 (p=0.997)	0.49 (p=0.148)	-0.38 (p=0.274)	0.16 (p=0.665)
IL-1β	0.92 (p=0.000)	0.90 (p=0.000)		0.55 (p=0.100)	0.85 (p=0.002)	0.53 (p=0.114)	IL-1β	0.07 (p=0.838)	0.00 (p=0.997)		-0.64 (p=0.045)	-0.02 (p=0.953)	-0.27 (p=0.444)
IL-10	0.35 (p=0.324)	0.31 (p=0.386)	0.55 (p=0.100)		0.26 (p=0.462)	0.24 (p=0.511)	IL-10	-0.27 (p=0.455)	0.49 (p=0.148)	-0.64 (p=0.045)		-0.53 (p=0.116)	0.26 (p=0.460
TGF-β	0.98 (p=0.000)	0.99 (p=0.000)	0.85 (p=0.002)	0.26 (p=0.462)		0.73 (p=0.017)	TGF-β	0.25 (p=0.493)	-0.38 (p=0.274)	-0.02 (p=0.953)	-0.53 (p=0.116)		-0.52 (p=0.122)
TNF-α	0.74 (p=0.015)	0.68 (p=0.030)	0.53 (p=0.114)	0.24 (p=0.511)	0.73 (p=0.017)		TNF-α	-0.28 (p=0.440)	0.16 (p=0.665)	-0.27 (p=0.444)	0.26 (p=0.460)	-0.52 (p=0.122)	

Supplementary Table S4. Correlation analysis of cytokines 6 months postoperatively (after GSLET) in patients with partial and total LSCD (pg/mL).

**Cytokine**	**Partial LSCD (n=5)**						**Cytokine**	**Total LSCD (n=6)**					
	IL-8	IL-4	IL-1β	IL-10	TGF-β	TNF-α		IL-8	IL-4	IL-1β	IL-10	TGF-β	TNF-α
IL-8		0.97 (p=0.000)	0.33 (p=0.348)	0.09 (p=0.815)	0.93 (p=0.000)	0.83 (p=0.003)	IL-8		-0.85 (p=0.002)	-0.27 (p=0.456)	-0.32 (p=0.367)	-0,35 (p=0.319)	0.09 (p=0.796)
IL-4	0.97 (p=0.000)		0.33 (p=0.351)	0.25 (p=0.479)	0.99 (p=0.000)	0.88 (p=0.001)	IL-4	-0.85 (p=0.002)		0.13 (p=0.712)	0.51 (p=0.132)	-0.14 (p=0.702)	-0.10 (p=0.781)
IL-1β	0.33 (p=0.348)	0.33 (p=0.351)		-0.42 (p=0.226)	0.32 (p=0.363)	0.16 (p=0.654)	IL-1β	-0.27 (p=0.456)	0.13 (p=0.712)		0.59 (p=0.072)	0.54 (p=0.105)	-0.82 (p=0.004)
IL-10	0.09 (p=0.815)	0.25 (p=0.479)	-0.42 (p=0.226)		0.32 (p=0.363)	0.42 (p=0.231)	IL-10	-0.32 (p=0.367)	0.51 (p=0.132)	0.59 (p=0.072)		-0.01 (p=0.976)	-0.48 (p=0.156)
TGF-β	0.930 (p=0.000)	0.99 (p=0.000)	0.32 (p=0.363)	0.32 (p=0.363)		0.89 (p=0.001)	TGF-β	-0.35 (p=0.319)	-0.14 (p=0.702)	0.54 (p=0.105)	-0.01 (p=0.976)		-0.32 (p=0.362
TNF-α	0.83 (p=0.003)	0.88 (p=0.001)	0.16 (p=0.654)	0.42 (p=0.231)	0.89 (p=0.001)		TNF-α	0.09 (p=0.796)	-0.10 (p=0.781)	-0.82 (p=0.004)	-0.48 (p=0.156)	-0.32 (p=0.362)	

Supplementary Table S5. Correlation analysis of cytokines 12 months postoperatively (after GSLET) in patients with partial and total LSCD (pg/mL).

**Cytokine**	**Partial LSCD (n=5)**						**Cytokine**	**Total LSCD (n=6)**					
	IL-8	IL-4	IL-1β	IL-10	TGF-β	TNF-α		IL-8	IL-4	IL-1β	IL-10	TGF-β	TNF-α
IL-8		1.00 (p=0.000)	-0.05 (p=0.890)	0.39 (p=0.267)	0.91 (p=0.000)	0.84 (p=0.002)	IL-8		0.67 (p=0.034)	0.22 (p=0.536)	0.31 (p=0.383)	0.82 (p=0.004)	0.03 (p=0.930)
IL-4	1.00 (p=0.000)		-0.04 (p=0.914)	0.38 (p=0.281)	0.90 (p=0.000)	0.85 (p=0.002)	IL-4	0.67 (p=0.034)		0.62 (p=0.057)	0.46 (p=0.177)	0.96 (p=0.000)	-0.08 (p=0.828)
IL-1β	-0.05 (p=0.890)	-0.04 (p=0.914)		-0.22 (p=0.534)	-0.15 (p=0.672)	0.04 (p=0.912)	IL-1β	0.22 (p=0.536)	0.62 (p=0.057)		0.58 (p=0.079)	0.61 (p=0.062)	-0.05 (p=0.892)
IL-10	0.39 (p=0.267)	0.38 (p=0.281)	-0.22 (p=0.534)		0.41 (p=0.241)	0.29 (p=0.409)	IL-10	0.31 (p=0.383)	0.46 (p=0.117)	0.58 (p=0.079)		0.49 (p=0.155)	0.14 (p=0.706)

TGF-β	0.91 (p=0.000)	0.90 (p=0.000)	-0.15 (p=0.672)	0.41 (p=0.241)		0.67 (p=0.035)	TGF-β	0.82 (p=0.004)	0.96 (p=0.000)	0.61 (p=0.062)	0.49 (p=0.155)		0.00 (p=0.994)
TNF-α	0.84 (p=0.002)	0.85 (p=0.002)	0.04 (p=0.912)	0.29 (p=0.409)	0.67 (p=0.409)		TNF-α	0.03 (p=0.930)	-0.08 (p=0.828)	-0.05 (p=0.892)	0.14 (p=0.706)	0.00 (p=0.994)	
